# *Streptococcus Oralis* meningitis from right sphenoid Meningoencephalocele and cerebrospinal fluid leak

**DOI:** 10.1186/s12879-019-4472-7

**Published:** 2019-11-11

**Authors:** Kishan Patel, Zain Memon, Adam Prince, Connie Park, Abin Sajan, Nazish Ilyas

**Affiliations:** Department of Internal Medicine, Zucker School of Medicine at Hofstra Northwell, Lenox Hill Hospital New York, New York, USA

**Keywords:** *Streptococcus oralis*, Meningitis, Sphenoid meningoencephalocele, Cerebrospinal fluid leak

## Abstract

**Background:**

*Streptococcus oralis* belongs to the *Streptococcus mitis* group and is part of the normal flora of the nasal and oropharynx (Koneman et al., The Gram-positive cocci part II: streptococci, enterococci and the ‘Streptococcus-like’ bacteria. Color atlas and textbook of diagnostic microbiology, 1997). *Streptococcus oralis* is implicated in meningitis in patients with decreased immune function or from surgical manipulation of the central nervous system. We report a unique case of meningitis by S*treptococcus oralis* in a 58-year-old patient with cerebral spinal fluid leak due to right sphenoid meningoencephalocele.

**Case presentation:**

A 58-year-old female presented in the emergency department due to altered mental status, fevers, and nuchal rigidity. Blood cultures were positive for S*treptococcus oralis*. Magnetic resonance stereotactic imaging of head with intravenous gadolinium showed debris in lateral ventricle occipital horn and dural thickening/enhancement consistent with meningitis. There was also a right sphenoidal roof defect, and meningoencephalocele with cerebrospinal fluid leak as a result. The patient was treated with ceftriaxone and had endoscopic endonasal repair of defect. She had complete neurologic recovery 3 months later.

**Conclusions:**

Cerebrospinal fluid leak puts patients at increased risk for meningitis. Our case is unique in highlighting S*treptococcus oralis* as the organism implicated in meningitis due to cerebrospinal fluid leak.

## Background

*Streptococcus oralis* is a member of *Streptococcus mitis* family and belongs to the *Viridans* group. The organism is part of the normal flora of the nasal, oropharyngeal, gastrointestinal, and genitourinary tracts [1]. The species usually presents with low pathogenicity and virulence [1]. There are case studies of *Streptococcus oralis* causing meningitis in individuals with decreased immune function and with anatomic manipulation of the central nervous system. These include cases caused by increased alcohol use [2], leukemia [3], spinal anesthesia [4], and neurologic surgeries [2]. *Streptococcus oralis* has caused meningitis in those with surgical manipulation of the dental cavity as well due to the anatomical location of the organism and propensity to cause meningitis in individuals [5, 6].

Cerebrospinal fluid leaks place patients at increased risk for meningitis. A retrospective study analyzing 111 patients with proven CSF leak from endoscopy, beta-2 transferrin, imaging, and/or fluorescein lumbar puncture had risk of meningitis of 19% over 12 years [7].. The most common organism implicated in meningitis from cerebrospinal fluid leaks is *Streptococcus pneumoniae* [7]. We present a unique case of acute meningitis due to *Streptococcus oralis* extending from sinusitis in a 58-year-old female with a right sphenoid meningoencephalocele. She had herniation of right temporal lobe through the sphenoidal roof defect with subsequent cerebrospinal fluid leak. MRI imaging of brain showed meningeal enhancement and ventricular debris, highly concerning for meningitis. CT head non-contrast showed herniation. Lumbar puncture was contraindicated. Guidelines from Infectious Disease Society of America in diagnosing bacterial meningitis include blood cultures and computed tomography scan of the head without contrast in select populations to evaluate for any contraindications to lumbar puncture [8]. Those with contraindications and high likelihood for meningitis are treated with targeted antibiotics based on blood culture results [8]. In these difficult diagnosis, MRI of the head can show meningeal enhancement and ventricular debris with high sensitivity and specificity [9].

## Case presentation

A 58-year-old female presented to the emergency department due to severe headaches, altered mental status with minimal response, neck stiffness, and fever of 38.3 C. She was not alert or oriented to person, place, or time. She was arousable with sternal rub and did track to visual stimuli. Per family members, these signs and symptoms started 1 day prior to admission. Her past medical history was significant for right sphenoid meningoencephalocele with herniation of the right temporal lobe through a defect in the roof of the sphenoid recess. This was never surgically addressed in the past. She never had trauma to the skull, tumors, or mass lesions in the brain before. She also had meningitis 10 years prior that was treated with an unknown antibiotic course as well as a history of sinusitis and clear nasal drainage that was worse with leaning forward for 3 months in duration. The patient denied sick contacts and recent travel. On admission (Day 0), her white blood cell count was 28.5 cubic milliliter (reference range 3.8–10.5 cubic milliliter), with a neutrophil predominance of 91%.

On day 0, 2 sets of anaerobic and aerobic blood cultures were obtained. Chlorhexidine was used as an antiseptic on the right antecubital region and venous blood was obtained for one set of aerobic and anaerobic blood cultures. A second set of blood cultures were taken within 1 h. Aerobic cultures were collected first with Aerobic Bactec plus culture vials from Becton, Dickinson, and Company. 10 ml were obtained for each bottle. Anaerobic cultures were collected second with Bactec lytic anaerobic culture vials by Becton, Dickinson, and Company. 10 ml were obtained for each bottle. There was no manipulation of the oral cavity prior to these culture sets. Urinalysis was negative for signs of urinary tract infection. Chest x-ray was negative for signs of pneumonia.

**Fig. 1:**
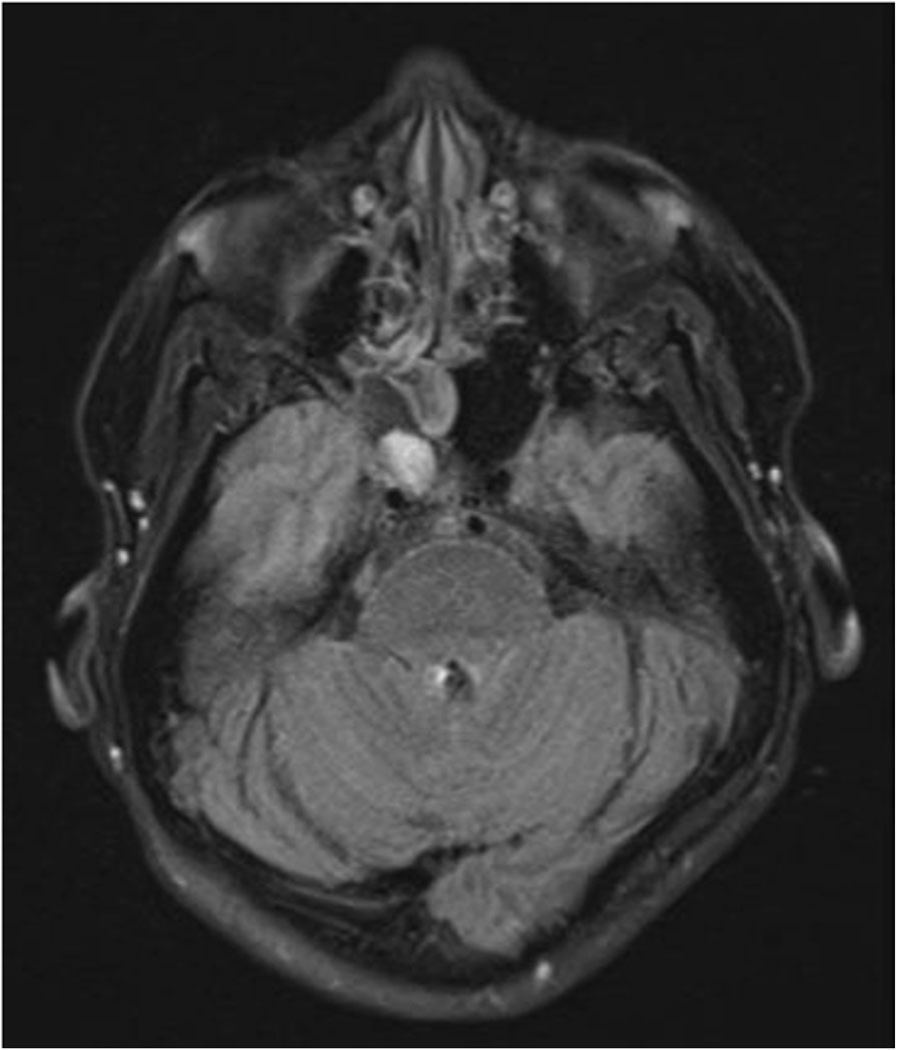
Magnetic resonance with intravenous gadolinium of the orbits face/neck coronal T2 turbo spin echo sinus demonstrated temporal lobe herniation through sphenoid roof defect

**Fig. 2:**
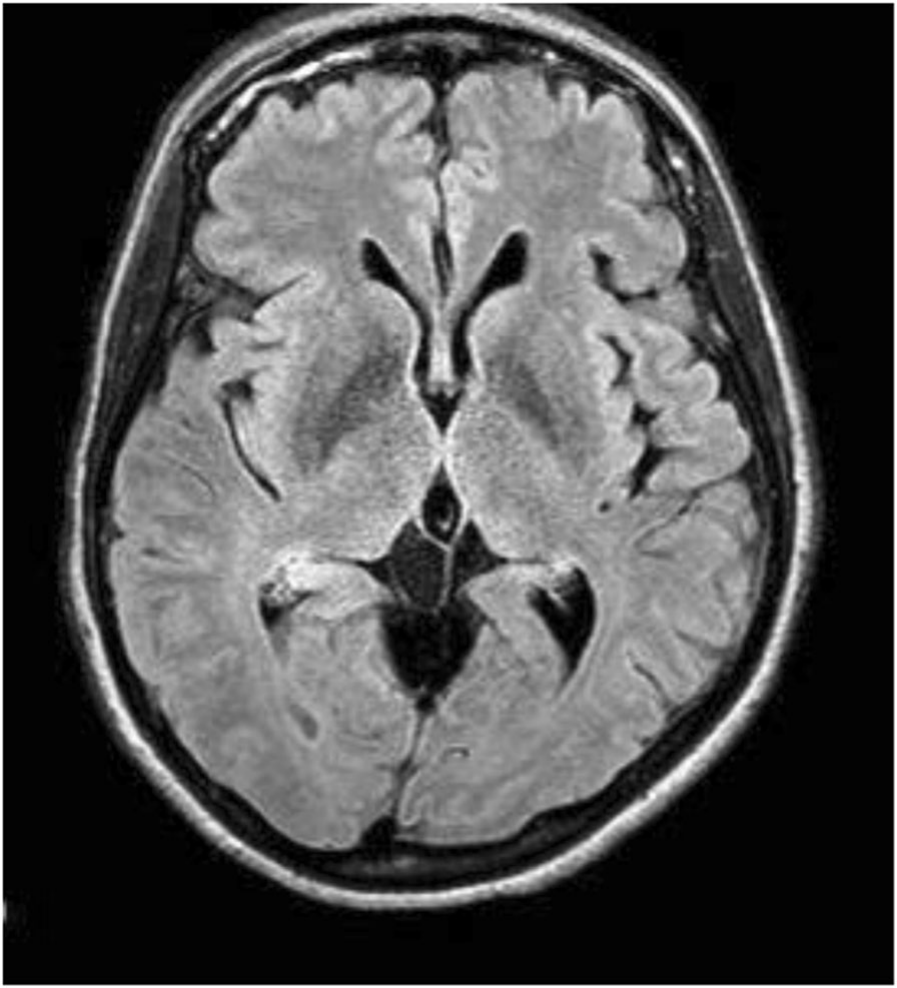
Magnetic resonance of head with T2-weighted-Fluid-Attenuated Inversion Recovery with intravenous gadolinium showing debris in the right lateral ventricle and dural enhancement consistent with meningitis

On day 0, MR of orbit, face, and neck with and without intravenous gadolinium contrast showed right sphenoid meningoencephalocele with herniation of the right temporal lobe as shown in Figs. 1 and 2. There was meningeal enhancement and ventricular debris present. Lumbar puncture was contraindicated secondary to prior herniation, but on day 7 magnetic resonance stereotactic imaging of head with intravenous gadolinium showed debris in lateral ventricle occipital horn and dural thickening/enhancement consistent with meningitis. Her neck stiffness, altered mental status, and fevers were also consistent with meningitis [8]. On admission, empiric coverage with vancomycin 1 g every 12 h, ceftriaxone 2 g every 12 h, and ampicillin 2 g every 6 h was initiated based on guidelines, in addition to dexamethasone 2 g every 2 h [8].

On day 1, both sets of aerobic and anaerobic culture bottles had giemsa staining that was positive for gram positive cocci in pairs and chains. All four culture bottles grew *Streptococcus oralis*. The isolate was susceptible to penicillin and ceftriaxone. On day 2, vancomycin and ampicillin were discontinued. By day 4, patient slowly regained mental status. On day 5, white blood cell count was 9.5 cubic milliliter (reference range 3.8–10.5 cubic milliliter). On day 5, beta 2 transferrin from nasal secretions were positive, confirming cerebrospinal fluid leak. Despite ceftriaxone therapy, patient continued to have low grade fevers from admission to day 4.

Transesophageal echocardiogram was performed to rule out endocarditis. No vegetations were observed. Otolaryngologist were consulted. On day 7, patient had endonasal endoscopic repair of roof of the sphenoid bone was performed. She improved clinically through her hospital course with resolution of mental status to her baseline. Three months later she had no complications as a result. Magnetic resonance imaging of brain without contrast after 1 month of treatment was negative for brain herniation, and patient remained afebrile without complication.

## Discussion and conclusions

Meningitis can be potentially devastating if not diagnosed and treated early. Patients with CSF leaks are at an increased risk for meningitis. This population can be difficult to diagnose meningitis if there are contraindications to lumbar punctures. Based on Infectious Disease Society of America, blood cultures should be collected first [8]. CT scan should be performed next, and if there are contraindications to lumbar puncture and a high clinical likelihood for meningitis, antibiotics must be administered as soon as possible [8]. In this scenario, antibiotics must target the organism collected form blood cultures [8].

Magnetic resonance imaging with intravenous gadolinium and fluid attenuated inversion recovery has high sensitivity and specificity for meningitis based on meningeal enhancement [9, 10]. *Streptococcus oralis* has not been implicated in meningitis from cerebrospinal fluid leaks in the literature. Risks of meningitis from chronic CSF leaks over 12 years are 19% and carry a high morbidity and mortality [7]. Beta 2 transferrin is a useful adjuvant in helping clinicians diagnose CSF leaks, with a sensitivity of 97% and specificity of 99% [11–13].

It is important for clinicians to suspect cerebrospinal fluid leak in those with altered mental status due to concern for spread of local organisms causing meningitis. Prompt antibiotics and surgical management are paramount in preventing morbidity and mortality [9]. It is important to rule out infective endocarditis because *Streptococcus oralis* has propensity to cause this disease burden [7]. Our case highlights that defects in the sphenoidal roof with meningoencephalocele can predispose patients to meningitis from *Streptococcus oralis*.

## Data Availability

The datasets used and/or analyzed during the current study are available from the corresponding author on reasonable request.

## Abbreviations

CSF: Cerebrospinal fluid
CT: Computed tomography
MR: Magnetic resonance
MRI: Magnetic resonance imaging
